# The Interaction of Human Papillomavirus Infection and Prostaglandin E_2_ Signaling in Carcinogenesis: A Focus on Cervical Cancer Therapeutics

**DOI:** 10.3390/cells11162528

**Published:** 2022-08-15

**Authors:** Janice García-Quiroz, Bismarck Vázquez-Almazán, Rocío García-Becerra, Lorenza Díaz, Euclides Avila

**Affiliations:** 1 Departamento de Biología de la Reproducción Dr. Carlos Gual Castro, Instituto Nacional de Ciencias Médicas y Nutrición Salvador Zubirán, Av. Vasco de Quiroga No. 15, Col. Belisario Domínguez Sección XVI, Tlalpan, Mexico City 14080, Mexico; 2 Departamento de Biología Molecular y Biotecnología, Instituto de Investigaciones Biomédicas, Universidad Nacional Autónoma de México, Av. Universidad 3000, Coyoacán, Mexico City 04510, Mexico

**Keywords:** cervical cancer, human papillomavirus, chronic inflammation, oncogenic proteins, cyclooxygenase-2, prostaglandin E_2_, cervical cancer treatment

## Abstract

Chronic infection by high-risk human papillomaviruses (HPV) and chronic inflammation are factors associated with the onset and progression of several neoplasias, including cervical cancer. Oncogenic proteins E5, E6, and E7 from HPV are the main drivers of cervical carcinogenesis. In the present article, we review the general mechanisms of HPV-driven cervical carcinogenesis, as well as the involvement of cyclooxygenase-2 (COX-2)/prostaglandin E_2_ (PGE_2_) and downstream effectors in this pathology. We also review the evidence on the crosstalk between chronic HPV infection and PGE_2_ signaling, leading to immune response weakening and cervical cancer development. Finally, the last section updates the current therapeutic and preventive options targeting PGE_2_-derived inflammation and HPV infection in cervical cancer. These treatments include nonsteroidal anti-inflammatory drugs, prophylactic and therapeutical vaccines, immunomodulators, antivirals, and nanotechnology. Inflammatory signaling pathways are closely related to the carcinogenic nature of the virus, highlighting inflammation as a co-factor for HPV-dependent carcinogenesis. Therefore, blocking inflammatory signaling pathways, modulating immune response against HPV, and targeting the virus represent excellent options for anti-tumoral therapies in cervical cancer.

## 1. Introduction

Cancer encompasses a group of complex diseases characterized by uncontrolled growth, evasion of anti-growth signaling, resistance to cell death, and colonization of distant niches by malignant cells. Cancer develops by a progressive multistep process from normal diploid cells, which are spontaneously or carcinogen-transformed to cancer cells. These initial cancer cells without a distinctive phenotype progressively evolve towards a malignant phenotype by the driving force of DNA alterations or genetic instability [1]. Several factors are involved in the origin of cancer including chemical carcinogenesis, radiation, air pollution, nutritional factors, and viral infection, among others. In this regard, infections by a virus such as Epstein–Barr, human herpesvirus 8, hepatitis B and C viruses, human T-lymphotropic virus type 1 and Merkel cell polyomavirus are linked to Hodgkin lymphoma and Burkitt lymphoma, Kaposi’s sarcoma, liver cancer, adult T-cell leukemia/lymphoma, and Merkel cell carcinoma, respectively [2]. Among causative viruses of cancer, human papillomaviruses (HPVs) are associated mainly with cervical cancer but also with neoplasias of the vulva, vagina, penis, anus, and oropharynx [2].

On the other hand, prostaglandin E_2_ (PGE_2_) is a lipid mediator of inflammation, which is one of the hallmarks of cancer [3]. Chronic inflammation promotes tumor development by enhancing survival, proliferation, and spreading of transformed cells while promoting tumor angiogenesis and evasion of tumor immune surveillance [3]. PGE_2_ bioavailability and signaling are highly regulated processes, and disruption of this regulatory axis is related to various human cancers, including cervical cancer [4]. This review is focused on the molecular mechanisms of carcinogenesis driven by HPVs and the participation of PGE_2_ signaling in cancer progression. Crosstalk between HPV infection and PGE_2_ is also addressed in this review. Finally, therapeutic options in cervical cancer based on HPV and PGE_2_ signaling are presented.

## 2. Cervical Cancer and Human Papillomavirus

Cervical cancer is by far the most clinically relevant HPV-related disease. In 2020, more than 600,000 women were diagnosed with cervical cancer worldwide, and about 342,000 women died. Accordingly, cervical cancer is the fourth most prevalent cancer among women globally, ranking after breast, colorectal, and lung cancer [5]. Although cervical cancer is still a disease with high mortality rates in developing countries, in high-income countries this type of cancer is one of the most preventable neoplasias, due to cytological screening of the cervix and prophylactic vaccination against HPV.

### 2.1. The Human Papillomavirus Life Cycle

HPVs are DNA viruses that probably represent the most prevalent sexually transmitted infection in both women and men worldwide. Based on their DNA sequence, more than 200 types of HPVs have been described; however, only a few HPV types are associated with health problems. HPVs are considered cutaneous or mucosal types according to their ability to infect epithelial cells of the skin or the inner lining of tissues. The last group of HPVs is important for cancer biology because they infect the lining of the mouth, respiratory tract, and anogenital epithelium. At least 20 HPVs are associated with lesions of the anogenital tract, which are further categorized as low-risk HPV (LR-HPVs types 6, 11, 42, 43 and 44, which cause benign warts) and high-risk HPVs (HR-HPVs types 16, 18, 31, 33, 34, 35, 39, 45, 51, 52, 56, 58, 59, 66, 68 and 70), which are associated with premalignant squamous lesions that are precursors of cancer [6]. HR-HPVs are detected in about 99% of cervical malignant lesions, HPV16 and HPV18 being responsible for at least 70% of all of them. Infection with an HR-HPV is necessary but not sufficient to cause cervical cancer because most HPV infections are efficiently cleared by the immune system without clinical disease.

HPVs are small non-enveloped double-stranded circular DNA viruses, in which only one of the DNA strands is transcribed. HPV DNA comprises approximately 8 kb organized into three functional regions: the early region encoding the non-structural proteins E1, E2, E4, E5, E6, and E7 that regulate viral gene expression and some of them, such as E1 and E2, also participate in DNA replication; the late region that encodes the structural proteins L1 and L2, needed for the viral capsid, and a long control region (LCR) that contains the regulatory sequences required for replication and transcription, namely, the origin of viral replication and the early promoter [7].

Although HPV replication requires E1 and E2 proteins, the participation of host proteins in this process are also required. HPVs infect only human epithelial cells, mainly keratinocytes. Cells located in the basal layer of the mucosa are the only cell type with the ability to divide and proliferate. This reservoir of basal cells is replenished by symmetric cell division of basal cells. However, asymmetric division of these cells generates two cells, one of them is used to renovate the basal cell population while the other one leaves the lower layer. Basal cells selected for terminal differentiation exit the cell cycle and stop DNA replication after they move out from the basal layer to the suprabasal layers. During this upward journey, cells acquire specialized properties and die when they arrive at the skin surface [8].

The HPV life cycle is closely linked with this renewal process occurring in the stratified squamous epithelium of the cervix. Because HPV infection is sexually transmitted, the viruses present in the anogenital region of HPV-infected men (mainly in the corona sulcus, glans, foreskin, and scrotum) are introduced in the inner female genital tract during sexual intercourse. HPVs gain access to the deepest layer via microabrasions or via hair follicles, finally reaching the single layer of epithelial cells located in the squamocolumnar junction between the endocervix and ectocervix. There, basal cells are infected by HPVs through the interaction of L1 capsid protein with heparan sulfate-containing proteoglycans which are thought to be the main HPV receptor [9]. In addition, keratinocyte-secreted laminin 5 [10] and cell adhesion α-6-integrin [11] also function as cellular co-receptors for HPVs. After specific binding on the keratinocyte cell surface, viral particles are slowly internalized by an actin cytoskeleton-dependent endocytic mechanism [12]. Inside basal cells, HPV capsid is disassembled, the viral genome is coated with L2 protein, packed inside transport vesicles, and delivered into the nucleus, whose envelope breaks down in early mitosis [13]. Like other DNA viruses, after mitosis and subsequent reformation of the nuclear membrane, the HPV genome-L2 protein complex harbored into the transport vesicles interact with promyelocytic leukemia nuclear bodies (also known as promyelocytic oncogenic domains), where transcription and replication take place [14,15].

In the initial phase of HPV replication, the viral genome in the form of episomes is maintained at a low copy number in infected basal cells [16]. HPV DNA replication requires the cooperation between the host DNA replication machinery and viral early proteins E1 and E2. HPV DNA replication starts with the binding of transcription factor E2 to specific sites located on LCR DNA, which results in the recruitment of E1 DNA helicase to the viral origin of replication, allowing the assembly of the DNA replication complex [17]. Initial infection with HPV is characterized by a low copy number of HPVs genomes (50–100 viral DNA copies per cell). An HPV persistent infection requires the production of a constant number of viral DNA in the nuclei of undifferentiated host cells during the cell cycle.

When HPV-infected basal cells move towards the epithelium surface, high levels of the HPV genome are synthesized, leading to the generation of progeny virions, which are sloughed from infected epithelia in the form of virion-laden squames. However, the HPV genome is often integrated into the host DNA in premalignant and malignant lesions of the cervix. HPV DNA integration represents a dead-end for the viral life cycle because infectious particles are no longer produced [6].

How does HPV maintain active DNA replication in arrested cell-cycle cells? HPVs have evolved successful strategies to actively replicate in infected growth-arrested host cells that are efficiently evading apoptotic signals. Among them, early proteins E5, E6, and E7 combined function are critical drivers of persistent viral infection and cellular transformation [18].

### 2.2. Mechanisms Underlying HPV-Driven Carcinogenesis

Unlike HPV E6 and E7 oncoproteins, little is known of the role of the E5 in the malignant transformation of the cervical epithelium. E5 is a multifunctional small hydrophobic polypeptide that is encoded by LR- and HR-HPVs and participates in key carcinogenesis points. Table 1 summarizes the role of high-risk HPV E5 on transformation, tumorigenesis, apoptosis, and immune modulation in cervical cancer. As noted, E5 oncoprotein contributes to cellular transformation driven by HPVs during the early stages of cervical carcinogenesis [19]. HPV DNA integration into the host’s genome correlates with the loss of E2, E5, and increased expression of E6 and E7 oncoproteins [19].

Both HR-HPV E6 and E7 are small proteins that cooperate together to keep HPV DNA replication and prevent apoptosis while inducing genome instability and immortalization of HPV-infected cells [7].

Transforming properties of HR-HPV E6 are closely linked to P53 activity, a tumor suppressor gene mutated in nearly half of human cancers [42]. P53 suppresses tumor development mainly by blocking the cellular proliferation of cells carrying damaged DNA and by induction of apoptosis in cancer cells. When DNA is damaged in proliferating cells, DNA repair is an essential process that prevents carcinogenesis. The lack of efficient DNA repair triggered by P53 and the suppression of apoptosis in HPV-infected cells are largely the main mechanisms of cellular transformation mediated by E6, resulting in damaged DNA accumulation and genomic instability [43].

**Figure 1:**
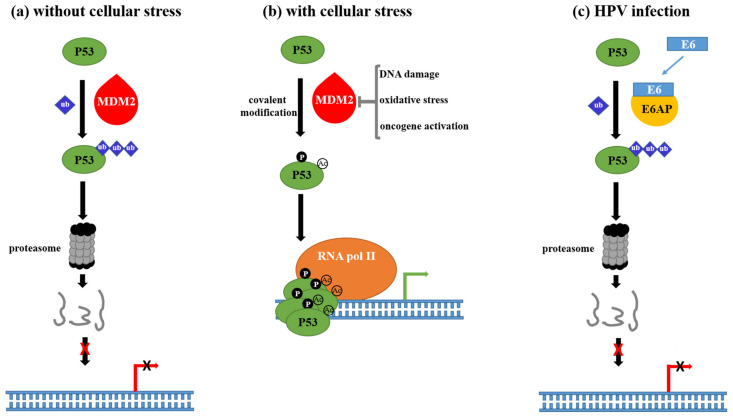
Role of P53 on normal cell physiology and during high-risk HPV infection. (**a**) In the absence of stressors, the coordinated action of MDM2 and proteasome maintain very low P53 bioavailability. (**b**) Stressor factors such as nutrient deprivation, genotoxic damage, and abnormal expression of oncogenes inhibit MDM2 activity while other cellular factors stabilize P53 by post-translational modification such as phosphorylation and acetylation. Acting as a homotetramer, P53 activates genes favoring DNA repair, apoptosis, and cell cycle arrest, among other processes involved in tumor development prevention. (**c**) Epithelial cells infected with HPV produce high levels of E6 oncogene, which binds E6AP. The complex E6AP-E6 targets P53 for degradation in the proteasome. Using this mechanism, E6 suppresses the protective response initiated by P53 against HPV infection. ub, ubiquitin; P, phosphorylation; Ac, acetylation.

Normal cells maintain very low levels of P53 protein through proteasomal degradation mainly by the action of E3 ligase murine double minute 2 (MDM2) [44] (Figure 1a). However, in response to genotoxic stress, several stimuli converge in the inhibition of MDM2, resulting in P53 accumulation in the nucleus. Activated P53, by both acetylation and phosphorylation, homotetramerizes and acts as a transcription factor that regulates genes involved in cell death, DNA repair, and cell cycle arrest, among others (Figure 1b). Hence, activation of the P53 pathway induces cell-cycle arrest at the G1 phase to provide an opportunity for cells to repair damaged DNA; however, when extensive DNA damage is detected, P53 activates apoptosis. HPV-infected cells cannot activate these anti-stress cellular processes because P53 protein levels are always insufficient (Figure 1c). HPV E6 binds to ubiquitin ligase E6-associated protein (E6AP), and this complex recruits P53, stimulating its ubiquitinylation and further degradation in the proteasome [45]. The P53 proteasomal degradation is not the only mechanism by which E6 abrogates P53 function. It was described that E6 reduces P53 transcriptional activity by targeting the coactivator CBP/p300 [46]. Thus, the presence of E6 on promoter regions switches CBP/p300 complex from an activating mode to a repressing state, leading to inhibition of P53-dependent transactivation [47]. Interestingly, in cells infected with types 5 and 8 HPV E6, the P53- and p300-dependent gene Ataxia Telangiectasia and Rad3-related (ATR), encoding for a phosphatidylinositol 3-kinase critical for the repair of UV-damaged DNA, is down-regulated [48].

Given the potential association of types 5 and 8 HPVs with non-melanoma skin cancer [49], it seems that E6 enhances the carcinogenic potential of UV radiation by down-regulation of both P300 and ATR and the concomitant accumulation of thymine dimers increasing DNA mutation rates.

HPV E6 is also required to extend the life span indefinitely in infected keratinocytes. Immortalization properties of E6 are achieved by a range of cellular proteins. Both abrogation of the retinoblastoma protein (pRB) pathway and overexpression of telomerase reverse transcriptase (TERT) are the drivers for the immortalization of normal keratinocytes [50]. It was described in cervical cells that the TERT promoter is activated by E6 from oncogenic HPVs [51], contributing in this way to the increase in TERT’s activity, needed for cervical carcinogenesis [52,53]. Another critical oncogene involved in the malignant transformation of the cervical epithelium is E7. This oncoprotein is an essential regulator of host transcription leading to cell cycle deregulation and immune evasion. E7 effects on host gene expression patterns are mediated by its interaction with several components of transcription and chromatin remodeling complexes, which are summarized in Table 2. Through these interactions, E7 exerts a broad impact on the host gene expression programs. Of particular importance is the interaction between E7 with members of the pRB family. pRB tumor suppressor is considered a key regulator of cellular processes such as cell cycle progression and apoptosis [54]. E7 targets pRB for proteasomal degradation [55], releasing the transcription factor E2F, which in turn activates genes needed for cell cycle progression into S-phase [56].

This mechanism and those summarized in Table 2 are responsible for the immortalization of HPV-infected cells and the induction of tumorigenesis.

Thus, early proteins E5, E6, and E7 encoded by HPVs play a pivotal role in the HPV life cycle and are required for the malignant transformation of the cervical epithelium by increasing host genomic instability and by blocking pivotal cell cycle checkpoints [18]. Other HPV proteins have a small contribution to cervical transformation. For instance, HPV16 E4 collapse the epithelial cell intermediate filament network in human keratinocytes by specific interaction with cytokeratins [71]. The cumulative sum of cellular alterations driven by HPV products leads to the onset of precancerous squamous lesions in the cervical tissue known as low-grade squamous intraepithelial lesions [LSIL, or cervical intraepithelial neoplasia grade I (CIN-I)], which can progress to high-grade squamous intraepithelial lesions [HSIL, a histological stage that encompasses the former entities CIN-2, CIN-3, moderate and severe dysplasia] and finally invasive squamous cell carcinoma [72]. Although the oncogenic role of HPV is well established, only a small number of HPV-infected women develop cervical cancer. Other cancer risk factors that promote persistent chronic HPV infection are cigarette smoking [73] and alcohol consumption [74]. Another cofactor for cancer development is chronic inflammation caused by HPV [75]. Chronic inflammation is considered a hallmark of human cancer, and proinflammatory factors are involved in cancer development [76].

## 3. Role of the Axis Cyclooxygenases/PGE_2_ and Its Receptors in Normal Physiology and Cancer

PGE_2_ exerts diverse physiologic and pathologic effects [77,78], regulating cellular processes in the immune [79], renal [80], cardiovascular [81], gastrointestinal [82,83], respiratory [84], and reproductive systems [85,86]. Additionally, PGE_2_ evokes important actions in bone metabolism [87], hematopoiesis [88], and is a crucial mediator of inflammation, fever, and pain [89,90,91,92].

### 3.1. PGE_2_ Biosynthesis and Metabolism

**Figure 2:**
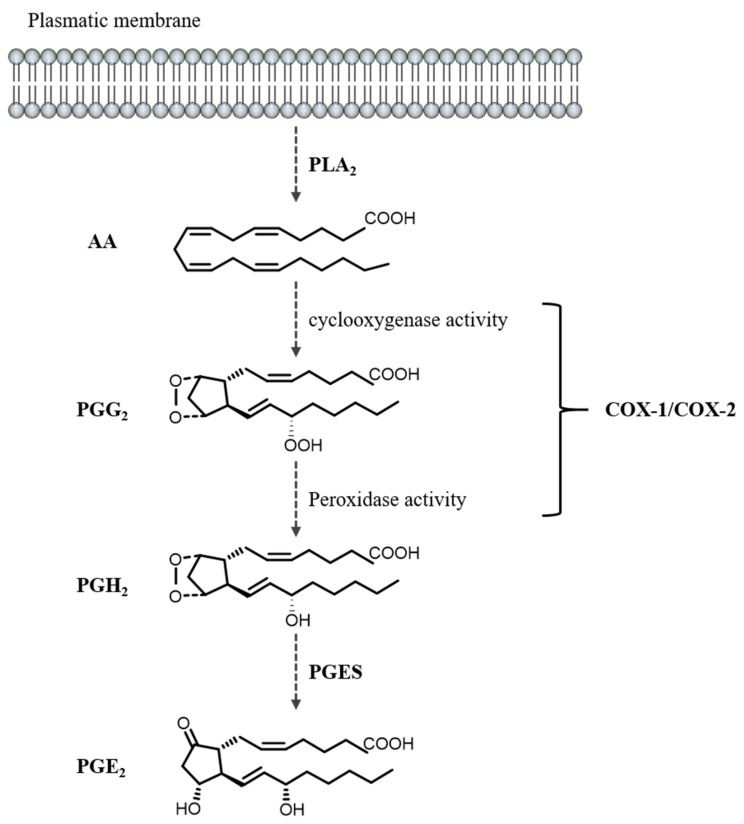
PGE_2_ biosynthesis. Arachidonic acid (AA) is released from membrane phospholipids by cytoplasmic phospholipase A2 (PLA_2_). The cyclooxygenase (COX) enzymes COX-1 and COX-2 convert AA to prostaglandin G2 (PGG_2_) and then prostaglandin H2 (PGH_2_). Subsequently, the enzyme PGE_2_ synthase (PGES) converts PGH_2_ to prostaglandin E2 (PGE_2_).

The prostaglandins (PGs) D2, E2, F2α, and I2 are eicosanoids that belong to the prostanoid family, which also includes thromboxanes (TXs) and prostacyclins. The basic chemical structure of prostanoids is a prostanoic acid with a cyclopentane ring and two carbon chains. The precursor molecule for these lipid mediators is arachidonic acid (AA), an essential polyunsaturated fatty acid that is stored in membrane phospholipids and released by the action of phospholipase A2 (PLA_2_) (Figure 2) [93,94,95]. Several signals activate PLA_2_, including phosphorylation by mitogen-activated protein kinase and an increase in intracellular calcium [96].

AA is afterward bis-dioxygenated by cyclooxygenase (COX) enzymes COX-1 and COX-2 (officially named prostaglandin endoperoxide synthases 1 and 2, respectively) to generate hydroperoxy endoperoxide PGG_2_, which is then reduced to the intermediate prostaglandin H2 (PGH_2_). COX-1 is constitutively expressed in most tissues and is responsible for the production of ‘housekeeping’ PGs that control a wide range of physiological effects, including maintaining homeostasis and gastrointestinal protection. On the other hand, COX-2 isoform expression is inducible at sites of inflammation and vascular trauma by a range of pro-inflammatory stimuli such as cytokines, growth factors, and ulcerogenic stimuli but particularly during infection and inflammation [97]. However, the COX-2 constitutive expression was also reported in selected organs in the absence of inflammation in the kidneys, gastrointestinal tract, and brain [98]. COX-2 expression is regulated mainly at the transcriptional level by inflammatory agents, cytokines, and growth factors [93].

Several specific isomerases and oxidoreductases convert PGH_2_ to the different types of PGs, such as PGE_2_, PGD_2_, PGI_2_, PGF_2α_, and TXA_2_ [93,94,95]. Biosynthesis of these metabolites depends on the tissue type and stimulus; specifically, PGE_2_, which is the most widely produced prostanoid in the body, is synthesized by PGE_2_ synthases (PGES) in a broad range of cell types [99]. After its synthesis, PGE_2_ is transported into the extracellular microenvironment by multidrug resistance-associated protein 4 [100,101]. PGE_2_ is metabolized mainly by nicotinamide adenine dinucleotide (NAD+)-dependent 15-hydroxyprostaglandin dehydrogenase to generate 15-keto-prostaglandin E_2_, which has significantly lower biological activity [102].

### 3.2. PGE_2_ Receptors

PGE_2_ performs its biological functions via interaction with four prostaglandin E receptor subtypes (PTGER1-4, also known as EP1-4) [103,104]. These receptors share around 30% sequence identity and belong to the G-protein-coupled, rhodopsin-type receptor superfamily [104]. PTGER1, PTGER2, PTGER3, and PTGER4 are located on human chromosomes 19, 14, 1, and 5, respectively [105,106,107]. The human PTGERs genes encode proteins of 402, 358, 390, and 488 amino acids with molecular masses of 42, 53, 43, and 53 kDa, respectively. PGE_2_ binds PTGER1, PTGER2, PTGER3, and PTGER4 with Kd values of 21, 40, 3, and 11 nM, respectively [104]. All of them exhibit differences in tissue distribution, signal transduction, and expression regulation [104,108]. PGE_2_ also has an affinity for other prostanoid receptors, such as the PGD_2_ DP1 receptor, and the PGF_2α_ FP receptor [109].

**Figure 3:**
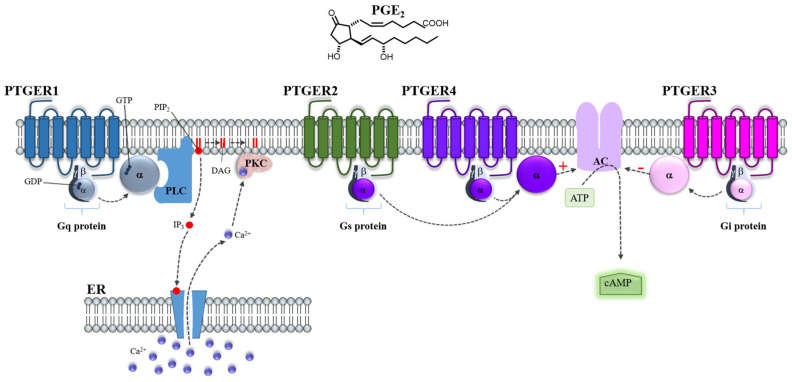
PGE_2_ activates four receptors, PTGER1-4. Prostaglandin E_2_ (PGE_2_) binds to G protein-coupled receptors identified as PTGER1-4. The binding of PGE_2_ to PTGER1 causes the exchange of guanosine diphosphate (GDP) for guanosine triphosphate (GTP) in the Gαq subunit allowing its dissociation from the βγ complex. The α subunit moves to phospholipase C (PLC) and activates it. This enzyme catalyzes the cleavage of the membrane phospholipid phosphatidylinositol 4,5-bisphosphate (PIP_2_) to produce two intracellular second messenger diacylglycerol (DAG) and inositol 1,4,5-trisphosphate (IP3). IP3 binds to specific calcium (Ca^2+^) channels releasing Ca^2+^ into the cytosol. Both IP3 and DAG contribute to activating protein kinase C (PKC). Regarding PTGER2 and PTGER4, the activation of adenylate cyclase (AC) by Gαs causes an increase in the intracellular cyclic adenosine monophosphate (cAMP) concentration formed from adenosine triphosphate (ATP). In contrast, the binding of PGE_2_ to PTGER3 causes inhibition of the activity of AC, resulting in diminished production of cAMP through the Gαi subunit. The activation of PTGER receptors regulates many cellular processes.

Upon being activated by PGE_2_, PTGER1 couples with G proteins and the Gαq subunit promotes the activation of phospholipase C (PLC), which hydrolyzes phosphatidylinositol 4,5-bisphosphate (PIP2) to diacylglycerol (DAG) and inositol 1,4,5-trisphosphate (IP3), leading to intracellular calcium mobilization and activation of protein kinase C (PKC) (Figure 3). Additionally, PKC activation induces desensitization of PTGER1, being PKC an important feedback regulator of the signal transduction of PTGER1 [110]. The PTGER1 activation also increases intracellular calcium mainly due to extracellular calcium influx through a pathway independent of PLC activation [110]. Both PTGER2 and PTGER4 are coupled to Gαs proteins to activate the adenylate cyclase (AC) enzyme increasing intracellular cyclic adenosine monophosphate (cAMP) levels (Figure 3) [103,104,111].

Even though fewer intracellular cAMP formation has been reported in cells expressing PTGER4 than in those expressing PTGER2 [112], the desensitization and internalization of PGE_2_-PTGER4 are faster than PGE_2_-PTGER2 [113].

PTGER2 and PTGER4 activate multiple signaling pathways; PTGER2, besides causing AMP production and protein kinase A (PKA) activation, increases the epidermal growth factor receptor (EGFR) and Src through its association with β-arrestin1, this leading to the subsequent activation of H-Ras/extracellular signal-regulated kinases (ERK), and phosphatidylinositol 3 kinase (PI3K)/protein kinase B (AKT) [114]. PTGER4 also activates PI3K, causing ERK phosphorylation [115]. PTGER2 is implicated in beneficial and adverse roles in the central nervous system, female reproduction, vascular hypertension, tumorigenesis, and peripheral diseases [116,117]. PTGER4 activation is associated with ductus arteriosus closure and inflammation-associated bone resorption [117].

PTGER3 activation induces its coupling to Gαi proteins, which inhibit AC decreasing intracellular cAMP concentration [103,104,111] (Figure 3). However, the splicing variants of the PTGER3 are coupled to different signaling pathways that act to both increase or decrease cAMP levels [104]. PTGER3 plays a crucial role in several biological events, such as fever, gastric mucosal protection, pain hypersensitivity, kidney function, and anti-allergic response [117]. Interestingly, PTGER3 expression is associated as a prognostic marker for cervical cancer [118].

### 3.3. Role of the COXs/PGE_2_/PTGERs Axis in Human Cancer

The axis COX-2/PGE_2_/PTGERs is involved in cancer progression through multiple pathways that regulate fundamental oncogenic process as cell proliferation, metastasis, angiogenesis, immune evasion, and cell death (Table 3).

Malignancies linked to HPV infection as cervical cancer, present high levels of COX-1/COX-2 and elevated synthesis of PGE_2_ [119,120]. In this regard, the incubation of HeLa cells with seminal plasma, which contains high levels of PGE_2_, up-regulated COX-2 expression and mRNAs of PTGER1, PTGER2, and PTGER4 [121]. Interestingly, seminal plasma alters vascular function by enhanced expression of angiogenic chemokines, such as interleukin (IL)-8, and growth-regulated oncogene alpha by mechanisms dependent on EGFR/ERK/COX and nuclear factor-kappa B (NF-κB) [122]. PTGER2 expression increases while PTGER3 expression decreases during cervical neoplasia development [118,123], suggesting these receptors might be considered as positive and negative prognostic markers of cervical cancer lesions, respectively. PTGER2 expression is a prognostic factor for the overall survival in the subgroup of negative PTGER3, and high galectin-3 expressed cervical cancer patients [124]. PTGER3 signaling promotes the migration of cervical cancer cells through the modulation of the urokinase-type plasminogen activator receptor [125]. Hence, the COX-2/PGE_2_/PTGERs axis plays an important role in the inflammatory environment seen in cervical cancer development.

**Table 3:** Participation of COX-2/PGE_2_/PTGERs axis in human cancer.

Cancer Type	COX-2/PGE2/PTGER1-4	Tumorigenic Role	Factors and Associated Genes	References
Colorectal	COX-2/PGE_2_/PTGER2	Angiogenesis	VEGF and Ang-2	[126,127]
Colon	COX-2/PGE_2_/PTGER2	Tumor microenvironment	CXCL1, IL6, WNT (2, 2B, 5A), MMP12	[128]
Gastric	COX-2/PGE_2_/PTGER4	Tumor microenvironment, metastasis	ADAM metalloproteases, EGFR ligands	[129]
	PTGER2/PTGER4	Cell growth inhibition		[130]
Cervical	PTGER2	Prognostic marker of disease		[123]
	COX-2/PGE_2_/PTGER3	Metastasis	uPAR	[125]
	COX-2/PGE_2_/PTGER4	Carcinogenesis		[32]
Lung	COX-2	Tumor microenvironment and inflammation	Cancer promoting cytokines	[131]
	COX-2/PGE_2_/PTGER4	Cell migration		[132]
	COX-2/PGE_2_/PTGER1	Cell proliferation and migration	ERK phosphorylation, β1 integrin activation	[133,134]
	COX-2/PGE_2_/PTGER3	Cell migration	MMP 2-9 VEGF, TGFβ, p-Smad 2-3	[135]
Breast	COX-2	Metastasis	MMP1	[136]
		Chemoresistance	MFGE8, KLK5, and KLK7	[137]
	PTGER3	Prognostic factor for progression-free survival		[138]
	COX-2/PGE_2_/PTGER2/ PTGER4	Angiogenesis, cell proliferation and stemness	MMP 2-9	[139,140]
	Nuclear PTGER1	Good prognosis marker		[141]
Bladder	COX-2	Stemness	Oct3/4, CD44v6	[142]
Vulva	COX-2/PGE_2_/PTGER4	Negative prognostic factor		[143]
Bone	COX-2	Cell migration		[144]
		Cell growth and progression, poor survival		[145]
Liver	COX-2	Activation of AKT and mTOR oncogenic pathways	AKT, TET1, MTOR, LTBP1, ADCY5 and PRKCZ	[146]
Prostate	COX-2/PGE_2_/PTGER4	Cell proliferation and migration	RANKL, RUNX2, MMP 2-9	[147]
Oral squamous carcinoma	COX-2/PGE_2_	Cell growth inhibition		[148]

## 4. Crosstalk between HPV Infection and PGE_2_/PTGERs Signaling on Cancer Progression

**Figure 4:**
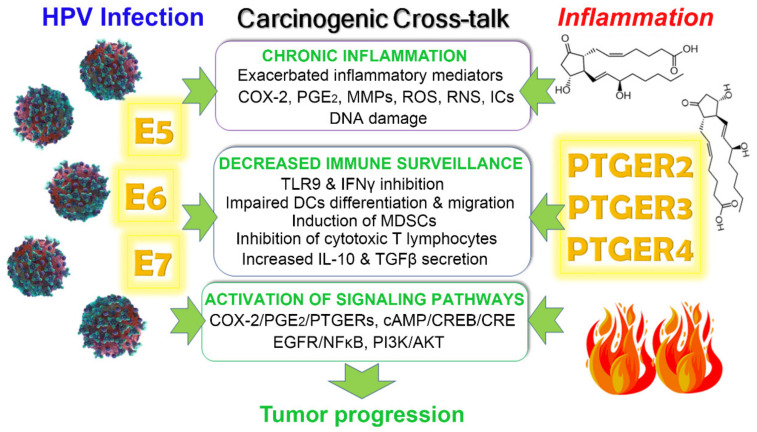
Crosstalk between HPV infection and PGE_2_ signaling on cancer progression. Cervical neoplasia may be promoted by HPV infection or chronic inflammation, processes that greatly interact to fuel tumorigenesis. HPV viral oncoproteins E5, E6 and E7 produce chronic inflammation by up-regulating COX-2 expression and consequently prostaglandin E2 (PGE_2_) production. HPV may also induce other inflammatory mediators such as reactive oxygen and nitrogen species (ROS, RNS), and PGE_2_ receptors (PTGER) expression. Likewise, PTGER activation results in increased expression/release of inflammatory cytokines (ICs) and metalloproteinases, as well as COX-2 activity/expression. Notably, ROS and RNS may cause DNA damage, facilitating HPV–DNA integration. Another important oncogenic mechanism in cervical HPV-dependent neoplasia is immune response evasion. Viral oncoproteins can drive dendritic cells (DCs) and lymphocytes towards a protolerogenic phenotype, inhibiting the expression of Toll-like receptor 9 (TLR9), down-regulating ICs such as interferon-gamma (IFNγ) while up-regulating immunosuppressive cytokines, including interleukin-10 (IL-10) and transforming growth factor beta (TGFβ), allowing HPV to survive. Furthermore, increased inflammatory mediators were associated with the induction of immunosuppressive myeloid-derived suppressor cells (MDSCs). Finally, known signaling pathways involved in the crosstalk between HPV infection and inflammation include PI3K/AKT, the epidermal growth factor receptor (EGFR)/nuclear factor-kappa B (NF-κB), COX-2/PGE_2_/PTGERs, and the cyclic AMP (cAMP)/cAMP-response element binding protein (CREB)/cAMP-responsive element (CRE).

A long-lasting infection can cause chronic inflammation, while inflammation by itself is responsible for causing as much as 25% of human cancers [149,150]. HPV may induce cancer through several mechanisms involving its viral oncoproteins, mainly E5, E6, and E7. These mechanisms produce a pro-tumorigenic environment that eventually can cause increased proliferation, inhibition of the host immune response, inactivation of tumor suppressor genes, mutations/immortalization, and malignant transformation, which are all considered hallmarks of cancer [151]. Notably, HPV carcinogenic mechanisms crosstalk with inflammatory pathways, favoring the tumorigenic process. Carcinogenic pathways of both the virus and inflammation intermingle at different points, involving distinct PGE_2_ receptors and viral oncoproteins that activate specific signaling pathways, as described in detail as follows and in Figure 4.

### 4.1. Chronic Inflammation

The knowledge that chronic inflammation can predispose an individual to develop cancer is now well established. A variety of factors can cause chronic inflammation, including bacterial infections, oxidative stress, chemical insults, and viral agents. In the case of HPV infection of neoplastic cervical epithelial cells, the viral oncoproteins E6 and E7 produce chronic inflammation by up-regulating COX-2 expression, and therefore PGE_2_ production [152]. The inductive effects of E6 and E7 were shown to be mediated by enhanced binding of activator protein-1 to the cAMP-responsive element (CRE) of the COX-2 promoter. E5 also up-regulates COX-2 expression but does so through EGFR/NF-κB signaling [31]. Moreover, the HPV E5 oncoprotein is known to induce the expression of the PTGER4 subtype in a cAMP-response element binding protein (CREB)/CRE-dependent pathway, potentiating in this way the proinflammatory activity of HPV [32]. Notably, HPV infection-dependent inflammation may generate reactive oxygen species (ROS) and nitrogen species (RNS), which can contribute to cancer initiation and progression by inducing DNA damage. Indeed, it was proposed that ROS and RNS generated by infection-related inflammatory processes have the potential to create DNA strand breaks, facilitating HPV–DNA integration, and contributing in this manner to carcinogenesis [153,154]. Therefore, inflammation and oxidative stress are widely interconnected, being free radicals an active component of both HPV infection and inflammation processes [149].

On the other hand, in the case of inflammation-induced carcinogenesis, known pro-inflammatory mediators involved in this process include matrix metallopeptidases (MMPs), cytokines such as tumor necrosis factor-alpha (TNFα), diverse interleukins, and COX-2. In particular, proinflammatory cytokines’ constant release activates signaling pathways that result in the formation of ROS and RNS [154]. Particularly in the setting of gynecological tumors, the COX-2-PGE_2_-PTGERs signaling is the central inflammatory pathway involved in carcinogenesis [155]. In this regard, PTGER2/PTGER4 PGE_2_ receptors are of clinical relevance in various tumors because of their involvement in stimulating COX-2 activity and/or expression and, consequently, PGE_2_ production [119], converging in this manner with the effects of E6 and E7 oncoproteins. Due to PGE_2_ being a recognized inflammatory prostaglandin, its role in maintaining chronic inflammation and creating an oncogenic environment is at sight. Indeed, inflammation is considered a cofactor in HPV-associated carcinogenesis [153]. PTGER2/PTGER4 mediate their effects on target cells via activating AC, increasing cAMP intracellular levels, which in turn activate the PKA pathway [121,155,156]. However, the PI3K/AKT signaling pathway was also linked to the PTGER2/PTGER4-triggered COX-2 transcriptional induction after PGE_2_ stimulation of cancer cells [157].

### 4.2. Immune Response Evasion

In cervical cancer, immune evasion is of the greatest importance for HPV to persist and cause malignant transformation. Viral oncoproteins and inflammation can both cause a local protolerogenic immune response, converging in the modulation of the activity of antigen-presenting cells (APCs), such as DCs, which can polarize the immune response. A tolerogenic immune response may help tumor cells and viral agents escape the immune system, allowing them to survive and grow [158]. In this regard, E6 and E7 oncogenes integrated into the host DNA of their target cells produce transcription products that inhibit TLR9 expression in DCs, causing their impaired differentiation, and consequently, down-regulation of immune surveillance. These HPV16 oncoproteins induce NF-κB to translocate into the nucleus, down-regulating TLR9 expression [159]. TLR9 down-regulation by HPV E7 oncoprotein was also demonstrated in HPV16-positive keratinocytes and cervical cell lines, through a mechanism that combines epigenetic and transcriptional events [160,161]. TLRs are expressed on both immune and nonimmune cells and are important for host defense activation because they can sense pathogen-derived products. Specifically, TLR9 is involved in sensing double-stranded DNA, such as that from HPVs. Furthermore, HPV can inhibit interferon (IFN) synthesis and its antiviral duties through E6 and E7 oncoproteins [162,163].

Notably, E6 was also described to inhibit the migration of DCs by increasing PGE_2_ in cervical lesions, which is another interconnecting point between HPV infection and PGE_2_ signaling [164]. In addition, E5 and E7 may block the activation of T lymphocytes, thus, helping infected cells expressing these oncoproteins to escape cytotoxic T-lymphocytes attack [165].

On the other hand, infected cancer cells per se also contribute to polarizing the tumor immune milieu, as it was found that supernatants from HPV-transformed cell cultures contained IL-10 and transforming growth factor (TGFβ), both immunosuppressive cytokines, while the analysis of HPV-positive cervical cancer biopsies showed a predominant immunosuppressive expression profile [158].

In a similar manner, in the case of inflammation, although counterintuitive to the proinflammatory activity of PGE_2_, this prostaglandin is known to promote immune tolerance through PTGER2 and PTGER3 receptors by inducing secretion of IL-10 in DCs [166]. Of note, upon PTGER2 and PTGER3 activation, the transcription factor CREB is phosphorylated. This suggests that both PTGER2 and PTGER3 induce cAMP mobilization, even though PTGER3 is typically associated with cAMP inhibition, as depicted in Figure 3. In addition, PTGER2 and PTGER4 are known to down-regulate the anti-tumor activity of PTGER-expressing natural killer cells, which are a type of cytotoxic T-lymphocytes (CTLs), by inhibiting their ability to kill tumor cells, cytokine synthesis, and chemotactic activity; thus, contributing to the pro-tumorigenic environment [167,168]. High PGE_2_ levels and other inflammatory mediators were also associated with stimulation of COX-2 expression in monocytes, contributing to the induction and persistence of immunosuppressive myeloid-derived suppressor cells (MDSC), which highlights the central role of COX-2/PGE_2_ signaling in tumor progression [169]. Indeed, MDSCs comprise a population of immature myeloid cells involved in suppressing antitumor immunity while stimulating cancer cell proliferation and tumor metastasis [170]. In particular, PGE_2_, via PTGER2 and PTGER4 expressed on MDSCs, inhibits the development of CTLs, impairing T cell functions [171]. As mentioned earlier, PGE_2_ is able to induce the rapid accumulation of cAMP, a process coupled with PTGER2/PTGER4 receptors [121]. This is very relevant to explaining the immunomodulatory effects of PGE_2_, given the well-known immunosuppressive effects of cAMP [172].

Considering this all together, it is clear that the COX-2-PGE_2_-PTGERs axis and cAMP/CREB signaling improve the oncogenic ability of HPV by promoting chronic inflammation in cervical cancer. In addition, the immunodepression caused by both HPV infection and inflammatory response is an additional pro-tumorigenic factor in cervical cancer progression. Thus, inflammation and immune modulation remain the main targets for therapies in cervical cancer.

## 5. Therapeutic Targeting of the COX/PGE_2_ Axis in Cancer

COX enzymes are the primary target of non-steroidal anti-inflammatory drugs (NSAIDs), which are widely used to relieve pain, fever, and other inflammatory processes. These drugs are classified according to their selectivity into non-selective NSAIDS and COX-2-selective NSAIDs [97].

### 5.1. The Non-Selective NSAIDS as Antineoplastic Agents in Cervical Cancer

The non-selective NSAIDs inhibit both COX-1 and COX-2 enzymes, and it was demonstrated that their long-term use may decrease the risk of colorectal, esophageal, breast, lung, prostate, liver, skin, and cervical cancers [173], which is in accordance to the previously discussed oncogenic role of inflammation. Regarding cervical cancer, few studies have reported this relationship, and most of them include the use of aspirin, whose long-term use is associated with decreased risk of cervical cancer [174]. Indeed, aspirin inhibits cervical cancer cell proliferation and colony formation in a time- and concentration-dependent manner, induces apoptosis, alters cell cycle distribution, and inhibits EGFR downstream cell survival signaling pathways [175,176,177]. The combined treatment of aspirin with radiation decreases cervical cancer cell proliferation to a greater extent than each condition alone and induces apoptosis by bcl-2 repression and caspase-3 induction [176]. Ibuprofen inhibits the growth and induces apoptotic cell death in cervical cancer cells while having no significant cytotoxicity on non-cancerous cells [175,178]. Meclofenolic acid exhibits great toxicity in cervical cancer both in vitro and in vivo, showing a significant reduction in tumor growth and increased survival of tumor-bearing mice. Other non-selective NSAIDs such as sulindac and indomethacin also inhibit cervical cancer cell proliferation and colony formation in a time and concentration-dependent manner; while nimesulide shows partial cytotoxicity [176,179].

### 5.2. COX-2 Selective NSAIDs as Antineoplastic Agents in Cervical Cancer

In addition to its typical therapeutical effects, some selective COX-2 inhibitors have chemopreventive and chemotherapeutic effects [180]. The mechanism of action of celecoxib to induce apoptosis could be independent of COX-2 inhibition, and rather the apoptosis may be due to the activation of caspase-8 and -9 with Bid cleavage and the loss of mitochondrial membrane potential, using as a possible target NF-κβ [181]. In a pilot study, it was determined that treating cervical cancer patients with celecoxib (400 mg twice daily/10 days) decreased the expression of COX-2, Ki-67, PGE_2_, and microvessel density in tumor biopsies [182]. Additionally, in a phase II randomized, double-blind placebo-controlled trial of celecoxib in patients with moderate and severe cervical dysplasia, the most of patients who received celecoxib had a good clinical response compared to the placebo group [183]. In another clinical trial carried out in patients with CIN-3 lesions and elevated serum vascular endothelial growth factor (VEGF) levels, the treatment with celecoxib showed greater regression than in the placebo group and decreased expression of COX-2 in cervical biopsies from treated patients was observed [184]. Moreover, the combinatorial effect of celecoxib with chemo- and radiotherapy have resulted in increased neoplastic cell sensitivity in different kinds of tumors [185,186], including patients with locally advanced cervical cancer in which the combination was associated with toxicity [187,188].

### 5.3. Corticosteroids in Cervical Cancer

Corticosteroids may inhibit the inflammatory process through modulating the transcription of anti-inflammatory and pro-inflammatory genes, including the down-regulation of COX-2 in monocytes and epithelial cells, so they are used to treat several inflammatory and immune diseases [189]. Taking into account the benefits of corticosteroids as anti-inflammatory agents in addition to their antiemetic effects, the synthetic corticosteroid dexamethasone was used as co-medication with radiotherapy in lymphoma and solid tumors [190]. Moreover, the corticosteroids protect cardiomyocytes from apoptosis induced by the cytotoxic doxorubicin, an antineoplastic drug known for its side effect of inducing cardiomyopathy [191]. While corticosteroids generally support therapy of lymphoid tumor cells, some studies describe interference of cancer therapy in cell lines of solid tumors. In this regard, dexamethasone inhibits cisplatin and 5-fluorouracil-induced apoptosis and promotes the growth of the majority of cells from solid tumors, but not of lymphoid cells [192]. Additionally, dexamethasone inhibits radiation-induced apoptosis in cervical carcinoma cell lines, which depends upon increased HPV E6 and E7; in contrast, the glucocorticoid had no effect on apoptosis of cells that either lack HPV or in which HPV E6 and E7 transcription is repressed by dexamethasone [193]. Part of the mechanism involved in dexamethasone-mediated proliferation and survival of cancer cells from solid tumors involves glucocorticoid receptor-mediated activation or suppression of target genes, modulation of the P53-dependent miR-145 expression in HPV-positive cervical cancer cells, and activation of HPV through the responsiveness of the upstream regulatory region (URR) of the virus [194,195]. The URR is responsible for the transcriptional regulation of the HPV major early promoter, which controls the expression of early genes of the virus, and interestingly, the URR of HPV18 is inducible by dexamethasone [195].

## 6. Targeting the Human Papillomavirus in Cancer

Epidemiological studies have shown the presence of HPV in up to 99% of cervical cancers and in a high percentage of other genital tract cancers [196]. Indeed, HPV is considered the main causative agent of genital tract cancers; offering a unique opportunity for infection prevention and treatment, which was addressed by the introduction of both prophylactic and therapeutic vaccines, among other treatments.

### 6.1. Preventing HPV Transmission Using Prophylactic Vaccines

Prophylactic vaccines are prepared using purified virus-like particles (VLPs) self-assembled by the major capsid protein L1, generating the recombinant proteins L1-VLPs, which are morphologically and immunologically similar to HPV virions, but that do not contain a viral genome. The LI-VLPs promote the generation of neutralizing antibodies directed against viral capsid proteins, blocking the adherence and internalization of HPV in the basal cells of the epithelium [197]. Several prophylactic vaccines were introduced into the market, including the bivalent, tetravalent, and nonavalent vaccines, named accordingly to the number of HPVs that can neutralize [196]. The bivalent vaccine is composed of the recombinant protein LI-VLPs of HPV16 and 18, the tetravalent contains LI-VLPs of HPV6, 11, 16, and 18, and the nonavalent is formed by LI-VLPs of HPV31, 33, 45, 52, 58, 6, 11, 16 and 18 [198]. Although prophylactic vaccines are effective in preventing HPV infection, they are not effective in eradicating it because they act by inducing neutralizing antibodies against L1 viral capsid protein, which is lost when HPV integrates into the host genome [196]. The problem that these vaccines have no therapeutic efficacy, the high demand for an efficient way to clear an established HPV infection, as well as the interest in the regression of precancerous and cancerous lesions have prompted the design of therapeutic vaccines [198].

### 6.2. Therapeutic Vaccines Targeting HPV Oncoproteins

Vaccines made against HPV oncoproteins may induce an immune response able to kill cervical cancer cells, representing a good therapeutic strategy and a promising treatment alternative to clear HR-HPV infections and associated malignancies. Among the proteins encoded by HPV, E6 and E7 are the main targets for the development of potential therapeutic vaccines due to: (1) their constitutive expression in infected cells, (2) both are functionally important for the development of tumor cells, and (3) they are readily recognized by the adaptive immune system as tumor antigens [196,198]. Other targets include E1, E2, and E5; however, their effectiveness is restricted because they are lost when the viral genome is integrated. Regarding HPV-therapeutic vaccines, several strategies were applied in their development, including the use of vectors (attenuated bacterial and viral)-based, nucleic acid-based, peptide/protein-based, and cellular-based vaccines [198].

#### 6.2.1. HPV-Therapeutic Vaccines Designed with Bacterial Vectors

Bacterial vector vaccines utilize attenuated bacteria to transport genes of interest into the cell. The bacterial vectors include *Lactobacillus casei*, *Listeria monocytogenes*, *Lactobacillus lactis*, *Lactobacillus plantarum*, and *Salmonella* species.

A recombinant *Lactobacillus casei* modified HPV16 antigen E7 vaccine completed the phase I/IIa clinical trial in patients diagnosed with CIN-3 positive to HPV16; interestingly, nine of these patients experienced disease regression to CIN-2, while five patients remarkably regressed to LSIL [198]. Another immunotherapeutic bacterial vector is *Listeria monocytogenes* (Lm), which is internalized via phagocytosis by APCs, where they can follow two routes. In the first, most bacteria are killed, providing a source of antigens for major histocompatibility complex (MHC) class II for activation of CD4+ helper T-cell. In the second, Lm being inside of phagolysosome, secretes the listeriolysin O (LLO), a pore-forming toxin that destroys the phagosomal membrane allowing it to escape and enter the cytosol, where it can be degraded by the proteasome and loaded onto MHC class I molecules for presentation to cytotoxic T cells [199]. The natural mechanism of infection of Lm was used to develop the HPV therapeutic vaccine, Lm-LLO-E7 (ADXS11-001 or AXAL), engineering by fusing HPV16 antigen E7 with a non-hemolytic fragment of LLO. The recombinant Lm secrets the tumor-associated antigens HPV16 E7 as fusion proteins with Lm antigens allowing both MHC class I and class II antigen presentation [196]. In a first study, mice xenografted with a cancer cell line expressing HPV antigen E6 and E7 were vaccinated with recombinant Lm expressing E7 alone or E7 as a fusion protein with LLO. Remarkably, in the last case, complete regression of 75% of tumors was achieved [200]. Afterward, numerous preclinical studies demonstrated Lm-LLO-E7 ability to induce regression of HPV-transformed tumors, leading to its advancement into multiple clinical trials [200]. The safety of Lm-LLO-E7 was evaluated in fifteen patients with metastatic, refractory, or recurrent invasive carcinoma of the cervix (ICC); the study showed that a live-attenuated Lm is safe to be administrated to late-stage ICC patients and a reduction in total tumor size was observed in four patients [201]. After the promising safe results of this clinical trial, multiple clinical trials followed, among them NCT02853604, an active phase III study of ADXS11-001 administered following chemo-radiation as adjuvant treatment for high-risk locally advanced cervical cancer, designed to compare the disease-free survival (study completion on October 2024, https://www.clinicaltrials.gov/ct2/results?cond=cervical+cancer&term=ADXS11-001+&cntry=&state=&city=&dist; accessed on 1 August 2022). The GTL001 vaccine was designed to induce a T-cell immune response to HPV16 and HPV18 and prevent the development of high-grade lesions in infected women who still have normal cervical cytology or minor abnormalities [202]. This vaccine was engineered by fusing HPV16 and HPV18 antigen E7 proteins with the catalytically inactive AC from *Bordetella pertussis* [203]. *Bordetella pertussis* secrets the AC-E7 protein fusion, which binds to the αMβ2 integrin receptor on the cell membrane of DCs and delivers its N-terminal catalytic AC domain into the cytoplasm, whose characteristic is used as the vector for antigen delivery into APC and subsequent antigen presentation [204].

#### 6.2.2. HPV-Therapeutic Vaccines Designed with Viral Vectors

Viral vectors are able to infect a variety of cell types and efficiently express encoded antigens, which makes them attractive candidates for the development of therapeutic HPV vaccines. Among the potential vectors evaluated in the development of therapeutic vaccines are vaccinia virus, adenovirus, adeno-associated virus and alphavirus [205].

Vaccinia virus is used to deliver E6 and E7 antigens for therapeutic HPV vaccination. Among the therapeutic vaccinia based-vaccine developed to treat genital cancers is the tissue-antigen HPV vaccine (TA-HPV), which expresses modified forms of E6 and E7 fusion proteins from HPV16 and HPV18, whose safety, immunogenicity, and efficacy were confirmed in patients with clinical early and late-stage of cervical cancer as well as vulval intraepithelial neoplasia and vaginal intraepithelial neoplasia [206,207,208]. The clinical benefits of this vaccine were enhanced by combining it with other therapeutic vaccines [209,210], local treatments such as surgery (NCT00002916), or immunomodulators (NCT00788164). Other vaccinia-based vaccine expresses LLO fused to HPV16 E7, which induces a strong immune response and causes the regression of established HPV16 immortalized tumors in C57BL/6 mice [211]. Modified Vaccinia Ankara (MVA) virus serves as a powerful vector system for developing vaccines against cancer, such as cervical cancer [212]. At this point, a recombinant vaccine expressing the MVA virus and the bovine papilloma virus E2 (MVA-E2) was created [213]. The orthotopic injection of MVA-E2 in murine models arrested tumor growth or promoted tumor regression [213,214]. Similarly, in clinical studies, direct injection of MVA-E2 virus particles in precancerous lesions of the cervix, anus, vulva, urethra, and uterus promoted their regression or complete elimination [215,216,217]. Additionally, the treated patients exhibited viral load reduction or even total clearance, and induction of long-lasting immune cytotoxic response correlating with no lesion recurrence [217]. Another recombinant MVA virus-based vaccine is MVA–HPV–IL2 (TG4001/R3484), which is engineered to express HPV16 E6, E7, and IL-2. The immunization of CIN-2/3 patients with this vaccine exhibited HPV16 clearance after 6 months of vaccination, and at 12 months, seven out of eight patients had no signs of CIN-2/3 relapse or HPV16 infection [218]. Likewise, the vaccination of mice with a replication-deficient adenovirus vector expressing calreticulin-HPV16 E7 fusion protein induced a stronger E7-specific immune response, inhibited tumor growth in pre-vaccinated mice xenografted with E7-expressing TC-1 cells, generating long-term memory against E7 and in established tumors resulted in complete tumor regression in all animals [219].

#### 6.2.3. Nucleic Acid-Based Vaccines against HPV

Nucleic acid vaccines are a method of immunization where DNA or mRNA sequences are delivered into the body to generate proteins, mimicking pathogens’ antigens to stimulate immune response [220]. VGX-3100 is a DNA-based vaccine consisting of two DNA plasmids containing codon-optimized sequences corresponding to the E6 and E7 genes of HPV16 and HPV18, whose safety, tolerability, efficacy, immunogenicity, and durability of response, were assessed in patients with HPV-related pre-cancerous conditions. VGX-3100 can drive the induction of robust immune responses to antigens from HR-HPV serotypes and could contribute to HPV16 and HPV18 elimination with complete histopathological regression of the dysplastic infectious process [221,222,223]. Other DNA-based vaccines targeting multiple HPV types are GX188E and pBI-1, which have completed phase I clinical trials exhibiting positive results regarding tolerance, CIN regression, and HPV clearance [224]. Regarding GX188E, it was demonstrated that electroporation-enhanced immunization with this vaccine, preferentially targeting HPV antigens to DCs, elicits a significant E6/E7-specific IFNγ producing a T-cell response in all evaluated CIN-3 patients [225,226]. Although different types of therapeutic vaccines exist, the best results have been achieved with MVA-E2, VGX-3100, GX-188E, and pBI-11. Particularly, MVA-E2 and VGX-3100 have reached phase III trials; however, their licenses are not yet authorized for marketing [224]. Other DNA-based vaccines candidates have also demonstrated a good safety and efficacy profile, including pNGVL4a-Sig/E7(detox)/HSP70 and pNGVL4a-CRT/E7(detox) [198].

#### 6.2.4. Peptide/Protein-Based Vaccines against HPV

Peptide-based vaccines have the potential to induce cellular immune responses mainly by the induction of cytotoxic T cells, with the capacity to recognize non-auto immunogenic cancer antigens eliminating malignant cells [227]. The peptide-based vaccine HPV-16-SLP consists of nine HPV16 E6 and four HPV16 E7 synthetic long peptides (SLP), whose immunogenicity and efficacy were investigated in HPV16-induced high-grade vulvar, vaginal, CIN, and pre-malignant disorders of the uterine cervix. Several clinical trials determined that the clinical responses to HPV-16-SLP correlated with the strength of HPV 16-specific T-cell immunity [228,229,230,231,232]. A clinical trial carried out in patients with HPV16 dependent vulval intraepithelial neoplasia (VIN) grade 3 exhibited increased clinical response according to the time elapsed after the last vaccination, and approximately 50% of them ended displaying a complete response, which correlated with kinetics and phenotype of induced T-cell responses [228,229]. Another peptide-based vaccine is PepCan, which consists of four synthetic peptides covering the HPV16 E6 protein and Candida skin test reagents as an adjuvant, whose safety and biological response was demonstrated in a clinical trial carried out in patients with CIN-2/3 lesions. This study demonstrated that the vaccine is safe while inducing a detectable immune response to E6 in 65% of the patients [233]. TA-CIN is a fusion protein vaccine that incorporates HPV-16 L2, E6, and E7, for the treatment of HPV16-associated genital disease. Vaccination of healthy volunteers with TA-CIN administrated without adjuvants reported no serious adverse events [234] and induction of L2-specific serum antibodies that neutralized several papillomavirus species [235]. The combination of TA-CIN with adjuvants elicited protective humoral and cell-mediated immunity to a greater extent than TA-CIN alone [236]. To date, a current pilot clinical trial is assessing the safety and feasibility of intramuscular administration of the TA-CIN vaccine as adjuvant therapy for patients with a history of HPV16-associated cervical cancer (NCT02405221).

These vaccines typically have increased stability during storage and transport as compared to DNA vaccines; however, they induce lower immunogenicity and poor stability in vivo, which remain their major drawbacks [227]. Additionally, other vaccines are currently under investigation, including DC-based vaccines, tumor cell-based vaccines, and T cell-based vaccines [205].

## 7. Immunomodulators

Imiquimod, an imidazoquinoline amine, is an immunomodulator approved for the treatment of external anogenital warts, small superficial basal cell carcinoma, and other malignancies [237,238]. Imiquimod activates innate and adaptive immune responses through activation of the TLR7, leading to inflammatory cell infiltration within the application area and apoptosis of diseased tissue [238]. The low patient compliance and high recurrence rate are significant problems for the treatment of genital warts by imiquimod; therefore, the combined use of this drug with therapeutic vaccines was proposed. Regarding this, a phase I clinical trial is currently studying the pNGVL4a-Sig/E7 (detox)/HSP70 DNA and TA-HPB vaccines side effects, the best dose scheme and clinical outcome when given with or without imiquimod in CIN-3 patients. The final data collection date for the primary outcome measure is expected in July 2023 (NCT00788164). In another phase I clinical trial, topic imiquimod was combined with GTL001 vaccine in patients infected with HPV16 or HPV18 with normal cytology, showing an acceptable safety profile and good antigen-specific cellular immune response [202]. In addition, a similar clinical trial determined that topical imiquimod with GTL001 vaccination induced antigen-specific CD8+ T cell responses leading to tumor regression in a murine model [203]. Co-administration of imiquimod with TA-CIN in VIN patients was investigated in a phase II clinical trial, showing significant infiltration of CD4+ and CD8+ T-cells in lesion responders at week 20 of treatment while complete lesion regression in 63% of patients was found at week 52 [239].

## 8. Chemical Antivirals

HPV DNA replication is an attractive target for HPV antivirals because its inhibition would result in fewer genomes available for viral protein synthesis and integration, a critical step in the development of HPV-dependent cancers [240]. Several studies and clinical trials have identified and demonstrated the robust anti-HPV potential of certain acyclic nucleoside phosphonates, antivirals targeting proteins encoded by HPV, and other host proteins utilized by HPV as targets of antiviral therapy [198].

### 8.1. Acyclic Nucleoside Phosphonates

Among the acyclic nucleoside phosphonates, cidofovir and adefovir are acyclic analogs of the corresponding monophosphates of deoxycytidine and deoxyadenosine. The active dephosphorylated forms of these antivirals are deoxynucleoside triphosphate analogs, potent inhibitors of viral DNA polymerases, reducing viral DNA synthesis [241]. Cidofovir has broad-spectrum antiviral against cytomegalovirus, herpes viruses, poxviruses, and papilloma viruses [241]. In HPV16 transformed keratinocytes lacking a viral DNA polymerase, cidofovir suppressed cell proliferation in a dose-dependent manner, induced DNA fragmentation, reduced P21 levels, and inhibited cell cycle progression in S-phase [242]. In cervical carcinoma and head and neck squamous cell carcinoma cell lines, cidofovir reduced E6 and E7 expression, induced P21, P53, and pRB accumulation, promoted the cell cycle arrest, reduced cyclin A, induced antiproliferative activity, triggered programmed cell death and radio-sensitized HPV-positive cells [243,244]. Cidofovir exhibited antiproliferative effects in HPV-positive and negative tumor cells, through its incorporation into DNA, causing its damage [245]. In vivo studies demonstrated significant tumor reduction with cidofovir in nude mice xenografted with HPV-positive cervical cancer cells [246]. Clinical trials have evaluated topical cidofovir in the treatment of patients with CIN and VIN, where the 60.8% and 46.1% of patients, respectively, were free of malignancy [247]. The antitumoral effect of cidofovir was also evaluated in combination with chemotherapy and radiotherapy, among others. In a phase I clinical trial carried out in patients with stage IB-IVA cervical cancer, it was determined that cidofovir (5 mg/kg/week) combined with chemo-radiotherapy appeared well tolerable and yielded significant tumor regressions [248]. On the other hand, some pro-drugs of adefovir and tenofovir showed significantly more antiproliferative activity against SiHa (HPV16), HeLa (HPV18), and C33A (HPV negative) cervical cancer cells, in comparison to their parent compounds [241].

### 8.2. Other Antivirals Targeting HPV Proteins Interaction

HPV-E1 and -E2 proteins are excellent targets for developing antivirals; on one side, HPV-E1 is an ATPase and helicase required for viral DNA replication, while on the other hand, E2 is required for transcription activation and repression. E2 recruits E1 at the beginning of replication, forming a ternary complex with viral DNA and the inhibition of this interaction blocks viral DNA replicating activity [17]. The indandiones are capable to inhibit E1–E2 protein interaction, acting by binding to the E2 N-terminal transactivation domain, the same protein region that interacts with E1. These antivirals display potent activity against E2 proteins of HPV6 and HPV11 [249]. Other compounds that inhibit HPV protein interaction are biphenylsulfonacetic acid and its derivatives, which are competitive inhibitors of the ATPase activity of HPV6 E1 helicase, inhibiting ATP hydrolysis by an allosteric mechanism involving tyrosine 486 [250].

## 9. Therapeutic Strategy against Cervical Cancer Using Nanoparticles and Gene Therapy

Nanotechnology is the approach to manufacturing nanoparticles, nanotubes, nanosheets, and nanorods, with a size from 1 to 100 nm in at least one dimension [251]. The development of nanotechnology drew attention to be used in medicine, with the idea of inserting nanorobots into patients to treat several diseases, including cancer [252]. The application of nanotechnology in cancer is named nano-oncology, which includes both diagnosis and therapeutic, employing nanomaterials that increase drug absorption, delivery, specificity, efficacy, and decreased side effects [252]. In cervical cancer, nanotechnology is being explored to enhance early diagnosis and improve treatment and vaccine efficacy; while HPV vaccines are usually delivered by intramuscular injection, nanotechnology offers a new approach for delivering vaccines by microneedles patch that efficiently delivers the vaccines into the epidermis and dermis region, which contain many Langerhans and DCs [251]. The microneedle array contains the vaccine antigen in the form of solution or suspension encapsulated in nanoparticles that induces a robust immune response [253]. Regarding this, in preclinical studies, the HPV vaccine Gardasil was intradermally delivered by Nanopatch, which comprises 10,000 micro projections/cm^2^ each 250 µm long, enhancing the antigenicity of the vaccine [254]. Additionally, in different preclinical studies, the efficacy of nanoparticles loaded with siRNA against E6 and/or E7 HPV oncoproteins was evaluated [255,256]. One of them assessed the efficacy of chitosan/HPV16 E7 siRNA nanoparticles, which were efficiently delivered into CaSki cervical cells and were observed to induce apoptosis [255]. Moreover, nanoparticles co-loaded with paclitaxel and E7 siRNA were developed with the aim to be simultaneously delivered to preclinical models of cervical cancer showing synergistic anti-tumor effect [257].

## 10. Conclusions

Standard treatments for advanced cervical cancer include surgery, radiotherapy, and chemotherapy, some of which are invasive and others have adverse side effects. In HPV-related malignancies such as cervical cancer, the COX-2–PGE_2_–PTGERs inflammatory signaling pathway, together with the cAMP/CREB/CRE cascade, are closely intermingled with the carcinogenic nature of the virus, highlighting inflammation as a co-factor for HPV-dependent carcinogenesis. Furthermore, the attenuation of the immune response caused by both the virus and inflammatory mediators represents an additional pro-tumorigenic process involved with cervical cancer development. Therefore, inflammation and immune modulation remain the main targets for anti-tumoral therapies in cervical cancer. In this regard, accessible therapeutic options for cervical cancer treatment and prevention have been developed in the last years. For instance, anti-inflammatory drugs constitute an excellent therapeutic strategy in cervical cancer patients. Indeed, it was suggested that some of these drugs may be re-purposed as antineoplastic agents, such as aspirin and celecoxib. On the other hand, HPV infection is considered the leading causative agent of genital tract cancers, representing an attractive therapeutic target for preventing and treating cervical cancer. Prophylactic vaccines against HPV made with recombinant DNA technology have been very effective in preventing persistent HPV infection and lesions that lead to cervical cancer. Highly immunogenic prophylactic vaccines induce a strong humoral immune response with the production of HPV-neutralizing antibodies that inhibit viral infection. The development of therapeutic vaccines for cervical cancer is based on activating cellular immunity against cancer cells instead of neutralizing antibodies. Among the different therapeutic vaccines developed against cervical cancer, the best results were achieved with MVA-E2, VGX-3100, GX-188E, and pBI-11. Particularly, MVA-E2 and VGX-3100 have reached the phase III trial; however, they have not been licensed for use yet. Additionally, the clinical benefits of HPV-therapeutic vaccines were enhanced by combining them with each other and local treatments or immunomodulators such as Imiquimod. The potency of therapeutic vaccines has been increased in preclinical studies employing nanotechnology, such as in the case of Nanopatch, which delivers a Gardasil prophylactic vaccine intradermally using microneedles. Finally, another way to target HPV is through antivirals, resulting in fewer genomes available for viral protein synthesis and integration, a critical step in the development of HPV-dependent cancers.

## Figures and Tables

**Table 1:** Summary of cancer-related processes triggered by high-risk HPV E5.

Process	References
HPV16 E5 triggers malignant transformation of murine keratinocytes	[20]
HPV16 E5 leads to cell growth in low serum and anchorage-independent growth of murine fibroblasts	[21]
HPV16 E5 stimulates the transforming activity of the epidermal growth factor receptor and lengthens receptor action by delaying its degradation	[22,23]
HPV16 E5 gene cooperates with E7 to stimulate cell proliferation and increases viral gene expression	[24]
HPV16 E5 enhances endothelin-1-induced keratinocyte growth	[25]
HPV16 E5 inhibits endocytic trafficking	[26]
HPV16 E5 impairs apoptosis in the early stages of viral infection in human keratinocytes	[27]
HPV16 E5 protects human foreskin keratinocytes from UV radiation-induced apoptosis	[28]
HPV16 E5 down-regulates surface HLA class I allowing persistent infection by avoiding host immune clearance	[29]
EGFR cooperates with HPV16 E5 to induce hyperplasia in mice	[30]
HPV16 E5 up-regulates COX-2 by a mechanism dependent on NF-kB and AP1	[31]
HPV16 E5 increases PTGER4 receptor for PGE_2_ in cervical cancer cells	[32]
HPV16 E5 represses the expression of stress pathway genes -XBP-1 and COX-2 in genital keratinocytes	[33]
HPV16 E5 synergizes EGFR signaling to enhance cell cycle progression and down-regulation of p27	[34]
HPV16 E5 inhibits apoptosis by proteasome-dependent degradation of Bax in human cervical cancer cells	[35]
Expression of HPV16 E5 produces enlarged nuclei and polyploidy in human keratinocytes	[36]
HPV16 E5 modulates the expression of host microRNAs miR-146a, miR-203, and miR-324-5p, and their target genes	[37]
HPV16 E5 induces switching from FGFR2b to FGFR2c and epithelial–mesenchymal transition	[38]
HPV18 E5 supports cell cycle progression and impairs epithelial differentiation by modulating EGFR signaling	[39]
HPV16 E5 increases MET, a growth factor receptor critical for tumor progression in human keratinocytes	[40]
HPV18 E5 cooperates with E6 and E7 in promoting cell invasion and in modulating the cellular redox state	[41]

**Table 2:** Some interaction partners of high-risk HPV E7 oncoprotein.

Protein Name (Symbol, Common Name)	Consequence of Interaction with E7	Reference
Cyclin-dependent kinase inhibitor 1B (CDKN1B, p27)	A cyclin-dependent kinase inhibitor. Inactivation of p27 by E7 promotes cell cycle S phase entry	[57]
Cyclin E1 (CCNE1, cyclin E)	A modulator of the cell cycle that functions as a regulatory subunit of CDK2. Enhanced kinase activity mediated by E7 interaction favors cell cycle G1/S transition	[58]
Cyclin-dependent kinase inhibitor 1A (CDKN1A, p21)	Another cyclin-dependent kinase inhibitor. E7 interaction with p21 promotes pRB phosphorylation by activated CDK2-cyclin A, enabling cell cycle progression	[59]
TATA-box binding protein (TBP, TFIID)	A critical factor in transcription initiation. Interaction between E7 and TBP participates in the transformation of epithelial cells	[60]
Proteasome 26S subunit, ATPase 4 (PSMC4, S4 subunit of the 26S proteasome)	An ATPase essential for protein turnover by the 26S proteasome. Upon interaction with E7, this protein might participate in pRB degradation by 26S proteasome favoring in this way the cell cycle progression	[61]
Retinoblastoma (pRB) RB transcriptional corepressor like 1 (RBL1, p107) RB transcriptional corepressor like 2 (RBL2, p130)	Hypophosphorylated pRB, p107, and p130 tumor suppressors inhibit E2F-mediated transcription initiation. Interaction of these proteins with E7 alleviates transcriptional inhibition promoting premature entry into the S-phase of the cell cycle	[62]
Fork head box M1 (FOXM1, fork head domain transcription factor MPP2)	A transcription factor involved in cell proliferation regulation. E7 enhances the transactivation and transformation properties of matrix metallopeptidase (MMP)-2	[63]
POU class 5 homeobox 1 (POU5F1, OCT4)	OCT4 is a transcription factor essential for stem cell pluripotency and embryonic development. E7 expression in differentiated cells stimulates OCT4 activity	[64]
Interferon regulatory factor 1 (IRF1, IRF-1)	A tumor suppressor gene with transcriptional regulation activity involved in immune responses. E7 direct inactivation of IRF1 promotes immune evasion of HPV in cancer	[65]
E1A binding protein P300 (EP300, Transcriptional coactivator P300)	A general transcriptional coactivator. By binding to P300, E7 impaired transcriptional regulation	[66]
Lysine acetyltransferase 2B (KAT2B, PCAF)	Another general transcriptional coactivator. E7 interaction reduces acetyltransferase activity impairing transcriptional regulation	[67]
Cyclin A2 (CCNA2, cyclin A)	A critical cell cycle regulator whose function activates cyclin-dependent kinase 2 (CDK2). E7 promotes cell cycle transition through G1/S and G2/M by activation of CDK2/cyclin A	[68]
E2F transcription factor 6 (E2F6, transcription factor E2F6)	E2F6 is a transcription factor that negatively regulates transcription. Interaction between E2F6 and E7 abrogates inhibitory action of E2F6, which extends the S-phase	[69]
Rho GTPase activating protein 35 (ARHGAP35, p190RhoGAP)	A GTPase activating protein for RhoA. Binding of E7 alters actin cytoskeleton dynamics and cell migration	[70]

## Data Availability

Not applicable.

## Abbreviations

AA	Arachidonic acid
AC	Adenylate cyclase
AKT	Protein kinase B
APC	Antigen-presenting cells
ATR	Ataxia Telangiectasia and Rad3-related
cAMP	Cyclic adenosine monophosphate
CIN	Cervical intraepithelial neoplasia
COX	Cyclooxygenase
CRE	cAMP-responsive element
CREB	cAMP-response element binding protein
CTL	Cytotoxic T-lymphocytes
DAG	Diacylglycerol
DC	Dendritic cells
EGFR	Epidermal growth factor receptor
ERK	Extracellular signal-regulated kinase
HPV	Human papillomavirus
HSIL	High-grade squamous intraepithelial lesion
IC	Inflammatory cytokines
ICC	Invasive carcinoma of the cervix
IFNγ	Interferon-gamma
IL	Interleukin
IP3	Inositol 1,4,5-trisphosphate
LCR	Long control region
LLO	Listeriolysin O
Lm	Listeria monocytogenes
LSIL	Low-grade squamous intraepithelial lesion
MDSCs	Myeloid-derived suppressor cells
MHC	Major histocompatibility complex
MMP	Matrix metallopeptidase
mTOR	Mammalian target of rapamycin
MVA	Modified Vaccinia Ankara
NF-κB	Nuclear factor-kappa B
NSAIDS	Non-steroidal anti-inflammatory drugs
PGE_2_	Prostaglandin E_2_
PGES	Prostaglandin E_2_ synthase
PI3K	Phosphatidylinositol 3-kinase
PIP2	Phosphatidylinositol 4,5-bisphosphate
PKA	Protein kinase A
PKC	Protein kinase C
PLA_2_	Phospholipase A2
PLC	Phospholipase C
PTGER	Prostaglandin E_2_ receptor
pRB	Retinoblastoma protein
RNS	Reactive nitrogen species
ROS	Reactive oxygen species
SLP	Synthetic long peptides
TERT	Telomerase reverse transcriptase
TGFβ	Transforming growth factor-beta
TLR	Toll-like receptor
TNFα	Tumor necrosis factor-alpha
TX	Thromboxane
URR	Upstream regulatory region
VEGF	Vascular endothelial growth factor
VIN	Vulval intraepithelial neoplasia
VLPs	Virus-like particles
